# Growth hormone alleviates oxidative stress and improves the IVF outcomes of poor ovarian responders: a randomized controlled trial

**DOI:** 10.1186/s12958-020-00648-2

**Published:** 2020-09-05

**Authors:** Yan Gong, Kun Zhang, Dongsheng Xiong, Jiajing Wei, Hao Tan, Shengfang Qin

**Affiliations:** 1 Reproductive Medicine Centre, Sichuan Provincial Women’s and Children’s Hospital, The Affiliated Women’s and children’s Hospital of Chengdu Medical College, #290 Shayan West Second Street, Wuhou District, Chengdu, Sichuan People’s Republic of China; 2 Department of Genetics, School of Bioscience and Technology, Chengdu Medical College, #783 Xindu Avenue, Xindu District, Chengdu, Sichuan 610500 People’s Republic of China; 3 Department of Medical Genetics and Prenatal Diagnosis, Sichuan Provincial Women’s and Children’s Hospital, The Affiliated Women’s and children’s Hospital of Chengdu Medical College, Chengdu, Sichuan People’s Republic of China

**Keywords:** Poor ovarian response, In vitro fertilization, Growth hormone, Oxidative stress, Reactive oxygen species

## Abstract

**Background:**

Oxidative stress (OS), defined as an imbalance between excessive reactive oxygen species (ROS) and/or reactive nitrogen species (RNS) production and antioxidant insufficiency, has been suggested to be involved in the pathogenesis of poor ovarian response (POR). Growth hormone (GH) can reduce OS in some cell types. This study investigated whether GH can improve OS and the in vitro fertilization and embryo transfer (IVF-ET) outcomes of poor ovarian responders.

**Methods:**

This study enrolled 105 patients with POR and 58 patients without POR (controls) who were diagnosed according to the Bologna criteria and underwent conventional IVF-ET. Poor ovarian responders were randomly assigned to two groups: the POR-GH group, which received pretreatment with GH 4 IU/d on day 2 of the previous menstrual cycle before IVF until the trigger day, and the POR-C group, which received no pretreatment. OS markers in follicular fluid (FF), ROS levels in granulosa cells (GCs), and the IVF outcomes of the groups were compared.

**Results:**

Endometrial thickness on trigger day, the number of cleaved embryos, the number of higher-quality embryos, and the rates of embryo formation, higher-quality embryo formation, implantation and clinical pregnancy were significantly increased in the POR-GH group compared with the POR-C group (*P* < 0.05). Moreover, compared to those in the non-POR group, FF malondialdehyde (MDA), total oxidant status (TOS), oxidative stress index (OSI) and ROS levels in GCs were significantly higher, whereas superoxide dismutase (SOD) and the total antioxidant capacity (TAC) were significantly lower in the POR-C group (*P* < 0.05). Furthermore, compared with those in the POR-C group, the FF TAC was significantly increased in the POR-GH group, and TOS, OSI and intracellular ROS levels were significantly reduced (*P* < 0.05).

**Conclusions:**

Pretreatment with GH alleviates OS and improves oocyte quality and IVF outcomes of poor ovarian responders.

**Trial registration:**

Chinese Clinical Trial Registry. ChiCTR1900021269. Registered 8 February 2019, http://www.chictr.org.cn/edit.aspx?pid=35837&htm=4.

## Background

Poor ovarian response (POR) remains one of the major challenges in woman infertility therapy including in vitro fertilization and embryo transfer (IVF-ET). Poor ovarian responders exhibit a higher cycle cancellation rate, produce fewer oocytes and cleaved embryos, and have a lower pregnancy rate and a higher miscarriage rate than individuals without POR [1]. The incidence of POR in IVF-ET ranges from 9 to 24% and is increasing as women delay childbirth [2]. The IVF outcomes of poor ovarian responders are still low despite the use of different stimulation protocols and multiple treatment courses [3].

Several factors, including advanced female age, ovarian and pelvic surgeries, chemotherapy and radiotherapy, are associated with POR [4]. The physiopathology of POR is complicated and includes follicular loss by atresia/apoptosis, diminished expression of follicle-stimulating hormone (FSH) receptor (FSHR), oocyte chromosomal defects and mitochondrial dysfunction [5, 6]. Accumulative evidence suggests that oxidative stress (OS) in ovarian ageing is an important pathogenesis of POR [6, 7]. Low to moderate levels of reactive oxygen species (ROS) and/or reactive nitrogen species (RNS) are involved in physiological processes, including defence against infections, cellular signalling systems, and cell growth and differentiation [8]. Excessive ROS/RNS may damage the innate antioxidant defence system and destroy proteins, DNA and lipids. OS is defined as an imbalance between excessive ROS/RNS production and a decrease in antioxidant defence systems in pathologic conditions [9].

Abnormal morphology, dysfunction and a low mitochondrial DNA (mtDNA) copy number have been observed in the oocytes of poor ovarian responders. Mitochondrial dysfunction results in a lower antioxidant production capacity, excessive ROS accumulation and DNA oxidative damage in oocytes, eventually contributing to poor embryo quality and IVF outcomes [10]. Therefore, antioxidant treatment may be beneficial for poor ovarian responders.

A meta-analysis reported that growth hormone (GH) supplementation could significantly improve the clinical pregnancy and live birth rates of poor ovarian responders [3]. The precise mechanism through which GH functions in poor ovarian responders is not fully understood, and most studies focus on its direct or indirect effects via growth hormone receptor (GHR) and insulin-like growth factor-I (IGF-I) [11]. Studies have revealed that GH augments the effects of gonadotropin on granulosa cells (GCs) and theca cells by binding to GHR and increasing the synthesis of IGF-I to improve follicle development and steroidogenesis [11, 12]. Moreover, Weall et al. [13] reported that GH increased the expression of GHR, improved mitochondrial function and increased the fertilization rate of oocytes among older women. GH can reduce OS in some types of cells [14–16]. However, the ability of GH to improve OS in poor ovarian responders has not been assessed in detail. Obtaining human oocytes for study is very difficult. Remarkably, the oocyte is supported and nourished by intimate cross-talk with its surrounding GCs [17]. The composition of follicular fluid (FF) reflects the metabolic and hormonal processes that occur in the microenvironment of the maturing oocyte [18]. Therefore, FF and GCs can be used as surrogate bioassays to study the biological processes of oocytes.

Based on the above evidence, this prospective, randomized, controlled study investigated the effects of GH on markers of OS in FF and GCs and the IVF outcomes of poor ovarian responders.

## Materials and methods

### Ethics statements

This prospective, randomized, open-label study was registered in the Chinese Clinical Trial Registry Centre (Registration No. ChiCTR1900021269) and approved by the Medical Ethics Committee of Sichuan Provincial Women’s and Children’s Hospital. Signed informed consent forms were obtained from all participants. All procedures in this study complied with the ethical standards of the relevant national and institutional committees on human experimentation and with the Helsinki Declaration 1975 (2013 revision).

The sample size calculation was based on the assumption that the clinical pregnancy rate would increase threefold after GH pretreatment. The clinical pregnancy rate of the poor ovarian responders in our centre was approximately 13%, and 48 patients were required for each group, with an α of 0.05 and a β error of 0.1 (power = 90%).

### Study subjects

Patients with POR (aged 33–43 years) diagnosed according to the Bologna criteria [1] who underwent IVF were enrolled from the Reproductive Medicine Centre of Sichuan Provincial Women’s and Children’s Hospital (between Feb. 2019 and Dec. 2019). The poor ovarian responders were randomized 1:1 to either GH pretreatment (the POR-GH group) or no GH pretreatment (the POR-C group) (using computer-generated random numbers). The exclusion criteria were as follows: (1) hydrosalpinx, congenital uterine malformations and/or endometrial disease such as tuberculosis or hyperplasia; (2) a basal follicle-stimulating hormone (bFSH) level ≥ 15 IU/L; (3) systemic lupus erythematosus, sicca syndrome, or polycystic ovarian syndrome; (4) an uncontrolled endocrinopathy such as diabetes, hyperthyroidism, hypothyroidism, or hyperprolactinemia; (5) IVF-ET treatment within three months; (6) an intracytoplasmic sperm injection (ICSI) cycle due to male infertility; or (7) supplementation with any antioxidants such as vitamin E, vitamin C, CoQ10, beta-carotene or selenium. Women with tubal factor infertility (aged 20–35 years) with a normal ovarian reserve and regular menstrual cycles who underwent IVF-ET were recruited as non-POR controls during the same period. The exclusion criteria for the non-POR group were the same as those for the POR group.

Data pertaining to the medical history of each participant were collected, including the regularity of menstrual cycles, the duration of infertility, and treatment history. Abnormal menstrual cycles included oligomenorrhoea, polymenorrhoea, irregular menstrual cycles, and amenorrhoea. Body mass index (BMI) was calculated as body weight divided by the square of body height (kg/m^2^). The antral follicle count (AFC) was determined by transvaginal ultrasound on days 2–3 of menstruation or progestin-induced bleeding withdrawal.

### Controlled ovarian stimulation (COS) and IVF

All patients underwent controlled ovarian stimulation (COS) with the GnRH antagonist protocol. Recombinant follicle-stimulating hormone (rFSH) (Gonal-F; Merck-Serono KGaA., Darmstadt, Germany) was injected on day 2 of the menstrual cycle, and rFSH doses were adjusted according to follicle growth and serum hormone levels. In the POR-GH group, 4 IU/d of recombinant human growth hormone (Changchun GeneScience Pharmaceuticals Co., Ltd., Changchun, Jilin, China) was injected subcutaneously on day 2 of the previous menstrual cycle before IVF until the trigger day (36–48 days). When a leading follicle reached 12 mm and/or serum E_2_ levels reached 300 pg/mL, Ganirelix (Merck Sharp & Dohme co., Ltd., Hoddesdon, United Kingdom) was administered. When at least one follicle was greater than 18 mm, recombinant human chorionic gonadotropin (hCG) (Ovitrelle®; Merck-Serono KGaA) was administered as the trigger. When no follicles with diameters ≥14 mm were noted after 10 days of gonadotrophin injection or when the peak E_2_ level was below 300 pg/mL, the cycle was cancelled. Ultrasound-guided transvaginal oocyte retrieval was performed 36 h later, and follicle flushing was not performed. Upon oocyte retrieval, FF was collected only from follicles with a diameter ≥ 16 mm measured on the retrieval day. FF samples were immediately centrifuged at 700 g for 5 min at room temperature, and the supernatant was stored at − 80 °C. The precipitates were suspended in 3 mL of PBS, gently layered in 3 mL 50% lymphocyte separation medium (Beijing Solarbio Science and Technology Corporation, Beijing, Solarbio Science and Technology Co., Ltd., China) and then centrifuged at 700 g for 10 min to remove red blood cells and debris. GCs layered at the interface of the gradient were washed twice with 5 mL of PBS (Nanjing KeyGen Biotech. Co., Ltd., Nanjing, Jiangsu, ChinaKeyGEN Bio TECH Co., Ltd., Jiangsu, China) and immediately examined for ROS using fluorescence microscopy and spectrophotometry. FF or GCs from each patient were collected separately and considered to be one sample. According to the criteria established by the Istanbul Consensus Workshop on Embryo Assessment, the cultured embryos on day 3 were assessed based on the number of blastomeres and the degree of fragmentation, and higher-quality embryos were categorized as grade A/B [19]. One or two fresh embryos with the best morphological grade were selected for transfer. The remaining embryos were all cultured to the blastocyst stage to be frozen. Luteal phase support with intramuscular injection of progesterone 60 mg/d was initiated on the oocyte retrieval day. Serum hCG was measured 12 days after ET, and hCG positivity was considered when hCG > 5 IU/mL. Clinical pregnancy was defined as demonstration of a gestational sac with an embryo showing cardiac activity. Early miscarriage was defined as loss of pregnancy before gestational week 12. Ovarian hyperstimulation syndrome (OHSS) was diagnosed according to Navot D et al. [20].

The maturation rate was calculated as the number of MII oocytes divided by the number of oocytes retrieved. The fertilization rate was calculated as the number of two pronuclear zygotes (2PN) divided by the number of MII oocytes. The embryo formation rate was calculated as the number of day 3 embryos divided by the number of 2PN. The higher-quality embryo formation rate was calculated as the number of higher-quality embryos divided by the number of 2PN. The implantation rate was calculated as the observed number of gestational sacs divided by the number of transferred embryos. The clinical pregnancy rate was expressed as the number of clinical pregnancy cycles divided by the number of embryo transfer cycles. The miscarriage rate was expressed as the number of spontaneous pregnancy loss cycles before 12 weeks of gestation divided by the number of clinical pregnancy cycles.

We suggest GH 4 IU/d pretreatment on day 2 of the previous menstrual cycle before IVF until the trigger day because a low physiological dose and longer treatment (from the antral follicle stage) might be more beneficial to follicular growth and development [2].

### Measurement of endocrine and metabolic parameters

Plasma glucose was measured using the hexokinase method (Beijing Strong Biotechnologies, Inc., Beijing, China). Oestradiol (E_2_), progesterone (P), total testosterone (TT), luteinizing hormone (LH), FSH, and insulin levels were measured using the electrochemiluminescence immunoassay platform (Roche Diagnostics GmbH, Mannheim, Germany). The serum level of anti-Mullerian hormone (AMH) was measured using an enzyme-linked immunosorbent assay kit (Guangzhou Kangrun Biotech, Co., Ltd., Guangdong, China). The homeostasis model assessment (HOMA-IR) index was calculated as fasting glucose (mmol/L) × fasting insulin (mU/L)/22.5 [21]. The intra- and inter-assay coefficients of the above variation were less than 5 and 10%, respectively.

### OS marker in FF assay procedures

FF malondialdehyde (MDA) concentrations (μmol/L) were measured using micro-MDA detection kits (NanJing Jiancheng Bioengineering Institute Co. Ltd., Nanjing, Jiangsu, China), and ultraviolet spectrophotometry (Shanghai Meipuda Instrument Co., Ltd., Shanghai, China) was performed at 532 nm. Superoxide dismutase (SOD) was determined using SOD kits (NanJing Jiancheng Bioengineering Institute Co. Ltd.) and spectrophotometry at 450 nm. The total antioxidant capacity (TAC) (mmol Trolox Equiv./L) and TOS (μmol H_2_O_2_ Equiv./L) were measured by the semiautomatic microplate colorimetric method using hydrogen peroxide (H_2_O_2_) and Trolox, respectively, for the standard [22, 23]. The oxidative stress index (OSI) was expressed as the ratio of TOS to the TAC. The measurements were performed in duplicate for each OS parameter. Serum samples from healthy volunteers were pooled for quality control. The intra- and inter-assay coefficients of the above variations were less than 5 and 10%, respectively.

### Detection of intracellular ROS levels

With a dichlorodihydrofluorescein diacetate (DCFH-DA) fluorescent probe, ROS levels in GCs were detected by ROS assay kit (Beyotime Biotechnology Co., Ltd., Shanghai, China). Briefly, GCs were resuspended in PBS and incubated with 10 μmol/L DCFH-DA in the dark for 25 min (37 °C) and then incubated together with 10 μg/mL 4′,6-diamidino-2-phenylindole (DAPI) (NeoFroxx, Frankfurt, Germany) for 5 min. After the cells were washed three times with PBS, GC suspensions were added to glass slides, and fluorescence was examined by fluorescence microscopy (Olympus Corporation, Tokyo, Japan). The examination wavelength was 488 nm, and the emission wavelength was 525 nm.

Similar to the above protocol but without DAPI, another set of GCs was used to measure the intracellular ROS level by NanoDrop UV-Vis spectrophotometry (Thermo Scientific, Massachusetts, USA). The fluorescence intensities are shown as the intensity in the POR group relative to that in the control group (non-POR group).

### Statistical analysis

All data were statistically analysed using SPSS 17.0 software (SPSS Inc., Chicago IL, USA). Continuous variables were expressed as the mean ± standard deviation (SD). The Kolmogorov–Smirnov test was used to assess the normality of the data distribution. Continuous variables with normal distributions were compared using the Student-Newman-Keuls test, and Bonferroni’s test was used as the post hoc test. Categorical data were compared using chi-squared tests. A two-tailed *P* ≤ 0.05 was considered statistically significant.

## Results

### Clinical, baseline endocrine and metabolic characteristics of the study population

**Fig. 1:**
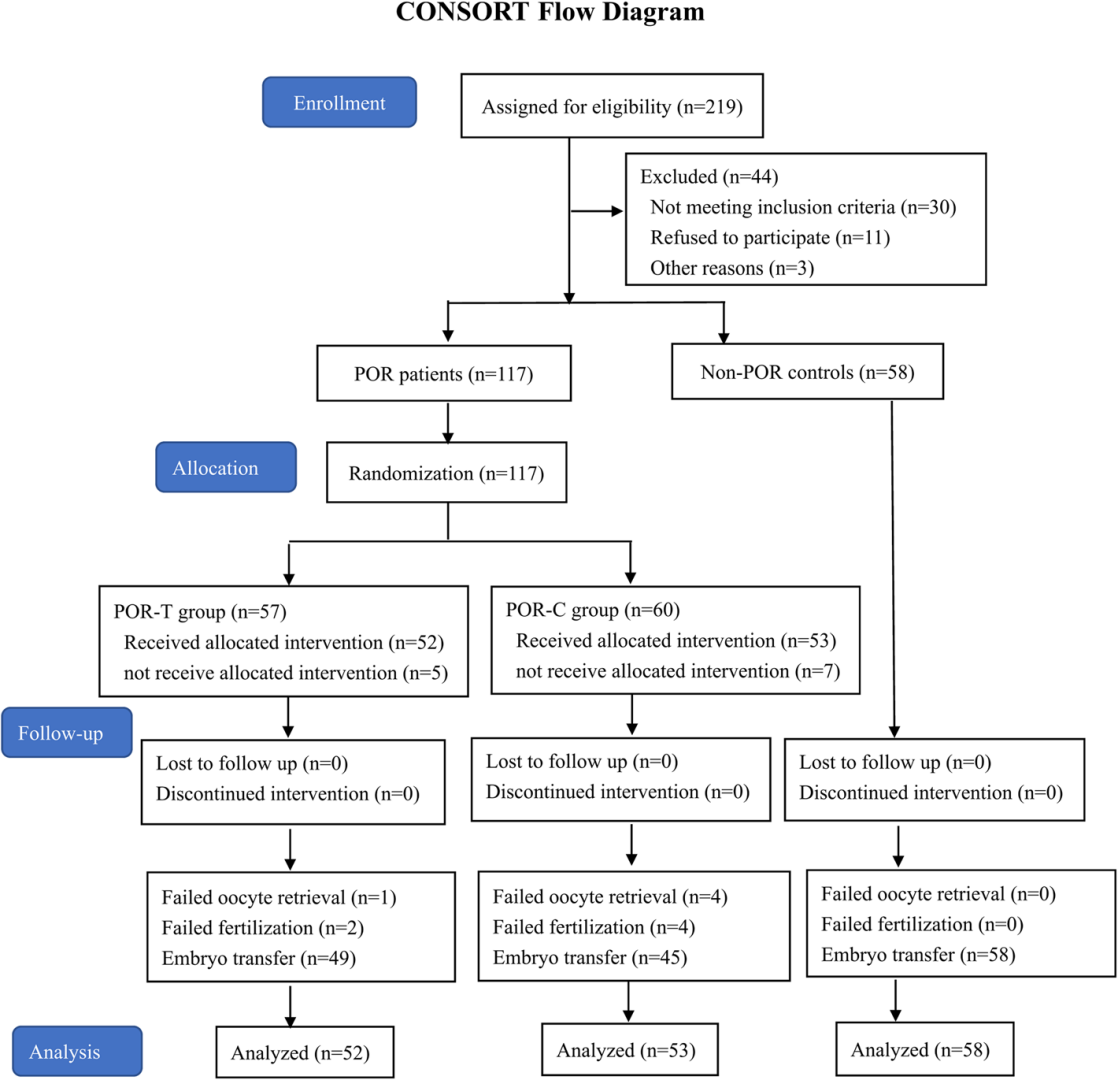
Flow diagram of this randomized controlled trial. Progression from recruitment to completion

The flow diagram of participant selection is shown in Fig. 1. Among poor ovarian responders, 38 had risk factors for POR (advanced age, abnormal menstrual cycles and ovarian surgery history), 44 had a previous POR three months prior (≤3 oocytes with a conventional stimulation protocol), and 71 had an abnormal ovarian reserve test (ORT) (AFC < 5–7 and/or AMH < 0.5–1.1 ng/mL). Compared to those in the non-POR group, age, basal FSH, the FSH/LH ratio and bE_2_ were significantly higher, whereas AFC and AMH were significantly lower in the POR-GH group and POR-C group (*P* < 0.05). The clinical, endocrine and metabolic characteristics did not significantly differ between the POR-GH and POR-C groups (*P* > 0.05) (Table 1).

**Table 1:** The clinical, basal endocrine and metabolic characteristics of the study population

	POR-GH (***n*** = 52)	POR-C (***n*** = 53)	Non-POR (***n*** = 58)
Age (years) ^ab^	38.41 ± 2.91	38.20 ± 2.79	29.56 ± 3.12
Infertility duration (years)	4.57 ± 3.14	4.39 ± 3.22	3.47 ± 2.22
Risk factors for POR cycle (n) ^ab^	20	18	0
Abnormal ORT cycle (n) ^ab^	39	32	0
Previous POR cycle (n) ^ab^	21	23	0
BMI (kg/m^2^)	22.09 ± 1.73	22.62 ± 2.73	22.19 ± 2.67
FSH (mIU/mL) ^ab^	11.49 ± 2.94	10.97 ± 2.75	5.89 ± 1.76
LH (mIU/mL)	4.59 ± 1.83	4.36 ± 1.57	4.29 ± 1.45
FSH / LH ratio ^ab^	2.74 ± 1.57	2.53 ± 1.68	1.38 ± 0.75
E_2_ (pg/mL) ^ab^	75.98 ± 34.05	72.34 ± 26.87	40.62 ± 11.19
P (ng/mL)	0.64 ± 0.39	0.54 ± 0.35	0.55 ± 0.20
TT (ng/mL)	0.37 ± 0.19	0.41 ± 0.20	0.43 ± 0.15
AMH (ng/mL) ^ab^	0.88 ± 0.67	0.81 ± 0.41	3.50 ± 1.91
PRL (μIU/mL)	162.59 ± 50.19	171.25 ± 52.32	163.51 ± 45.79
HOMA-IR	1.68 ± 1.25	1.71 ± 1.34	1.67 ± 1.33
AFC ^ab^	4.12 ± 1.71	4.06 ± 2.11	15.30 ± 4.56

**Note:** Data are presented as the mean ± SD or number. *BMI* body mass index, *FSH* follicle-stimulating hormone, *LH* luteinizing hormone, *E*_*2*_ oestradiol, *P* progesterone, *TT* total testosterone, *AMH* anti-Mullerian hormone, *PRL* prolactin, *HOMA-IR* homeostatic model assessment of insulin resistance, *AFC* antral follicle count

^a^ *P* < 0.05 the POR-GH group versus the non-POR group

^b^ *P* < 0.05 the POR-C group versus the non-POR group

### COS and IVF outcomes were improved in the POR-GH group

In the POR-GH group, 3 cycles were excluded because no oocytes were retrieved or because fertilization failed. In the POR-C group, 8 cycles were excluded because of the absence of follicular growth, failed oocyte retrieval or failed fertilization. Compared to those in the non-POR group, the rFSH duration, E_2_ levels on trigger day, the number of retrieved oocytes, the number of MII oocytes, the number of fertilized oocytes, the number of cleaved embryos and the number of higher-quality embryos were significantly lower in the POR-GH group and POR-C group, but the rFSH doses were significantly higher (*P* < 0.05). The endometrial thickness on trigger day, implantation rate and clinical pregnancy rate were significantly reduced, whereas the excluded cycle rate was significantly increased in the POR-C group compared with the non-POR group (*P* < 0.05). Endometrial thickness (9.65 ± 1.84 vs. 8.61 ± 1.23 mm), the number of cleaved embryos (2.31 ± 1.81 vs. 1.73 ± 1.03), the number of high-quality embryos (1.26 ± 0.65 vs. 0.72 ± 0.56), the embryo formation rate (95.76% vs. 83.87%), the higher-quality embryo formation rate (52.54% vs. 35.48%), the implantation rate (28.21% vs. 9.72%) and the clinical pregnancy rate (38.77% vs. 13.33%) were significantly increased in the POR-GH group compared with the POR-C group (*P* < 0.05). No side effect or moderate or severe OHSS was reported in the study population (Table 2).

**Table 2:** Controlled ovarian stimulation, IVF outcomes and OS markers in FF

	POR-GH (***n*** = 52)	POR-C (***n*** = 53)	Non-POR (***n*** = 58)
rFSH doses (IU) ^a,b^	2491.46 ± 996.47	2499.87 ± 1345.16	1875.09 ± 467.39
rFSH duration (d) ^a,b^	8.93 ± 2.57	8.97 ± 4.11	10.65 ± 1.65
E_2_ on trigger day (pg/mL) ^a,b^	985.13 ± 348.44	887.85 ± 372.21	1956.10 ± 558.48
Endometrial thickness (mm) ^b,c^	9.65 ± 1.84	8.61 ± 1.23	10.33 ± 1.93
Oocytes retrieved ^a,b^	3.71 ± 2.50	3.24 ± 2.56	12.51 ± 6.81
MII oocytes ^a,b^	3.18 ± 1.78	2.87 ± 1.89	9.95 ± 4.28
Fertilized oocytes (2PN) ^a,b^	2.36 ± 1.86	2.02 ± 1.21	7.04 ± 4.29
Cleaved embryos ^a,b,c^	2.31 ± 1.81	1.73 ± 1.03	5.38 ± 3.62
Higher-quality embryos ^a,b,c^	1.26 ± 0.65	0.72 ± 0.56	3.34 ± 2.78
Oocyte maturation rate (%)	82.38% (159/193)	83.02% (132/159)	79.48% (577/726)
Oocyte fertilization rate	74.21% (118/159)	70.45% (93/132)	70.71% (408/577)
Embryo formation rate ^a,c^	95.76% (113/118)	83.87% (78/93)	76.47% (312/408)
Higher-quality embryo formation rate ^b,c^	52.54% (62/118)	35.48% (33/93)	47.55% (194/408)
No. ET	1.58 ± 0.51	1.59 ± 0.48	1.43 ± 0.50
Excluded cycle rate (%) ^b^	5.77% (3/52)	15.09% (8/53)	0/58
Implantation rate (%) ^b,c^	28.21% (22/78)	9.72% (7/72)	43.37% (36/83)
Clinical pregnancy rate (%) ^b,c^	38.77% (19/49)	13.33% (6/45)	53.44% (31/58)
Miscarriage rate (%)	21.05% (4/19)	33.33% (2/6)	6.45% (2/31)
Multiple pregnancy rate (%)	15.78% (3/19)	16.67% (1/6)	16.13% (5/31)
In FF			
MDA (nmol/mL) ^a,b^	2.27 ± 0.69	3.02 ± 1.10	1.33 ± 0.67
SOD (U/mgprot) ^a,b^	12.99 ± 2.32	11.21 ± 1.71	14.76 ± 1.93
TAC (mmol Trolox Equiv./L) ^a,b,c^	0.59 ± 0.13	0.42 ± 0.16	0.71 ± 0.11
TOS (μmol H_2_O_2_ Equiv./L) ^a,b,c^	8.06 ± 1.19	10.14 ± 4.86	5.67 ± 1.09
OSI ^a,b,c^	16.49 ± 8.19	23.87 ± 10.13	8.16 ± 1.86

**Note:** Data are presented as the mean ± SD or percentage (number). *2PN* Number of two pronuclear zygotes, *ET* embryos transferred, *FF* follicular fluid, *MDA* malondialdehyde, *SOD* superoxide dismutase *TAC* total antioxidant capacity, *TOS* total oxidant status, *OSI* oxidative stress index. A chi-squared test was performed to compare the rates of cycle cancellation, implantation, clinical pregnancy, miscarriage and multiple pregnancy

^a^ *P* < 0.05 the POR-GH group versus the non-POR group

^b^ *P* < 0.05 the POR-C group versus the non-POR group

^c^ *P* < 0.05 the POR-GH group versus the POR-C group

### The levels of OS markers in the FF of the POR-GH group were decreased

Compared to those in the non-POR group, MDA, TOS and OSI levels in FF were significantly higher in the POR-GH group and POR-C group, but the levels of SOD and TAC were significantly lower (*P* < 0.05). The TAC (0.59 ± 0.13 vs. 0.42 ± 0.16) was significantly increased, whereas TOS (8.06 ± 1.19 vs. 10.14 ± 4.86) and the OSI (16.49 ± 8.19 vs. 23.87 ± 10.13) were significantly decreased in the POR-GH group compared with the POR-C group (*P* < 0.05) (Table 2).

### ROS accumulation in GCs of the POR-GH group was inhibited

**Fig. 2:**
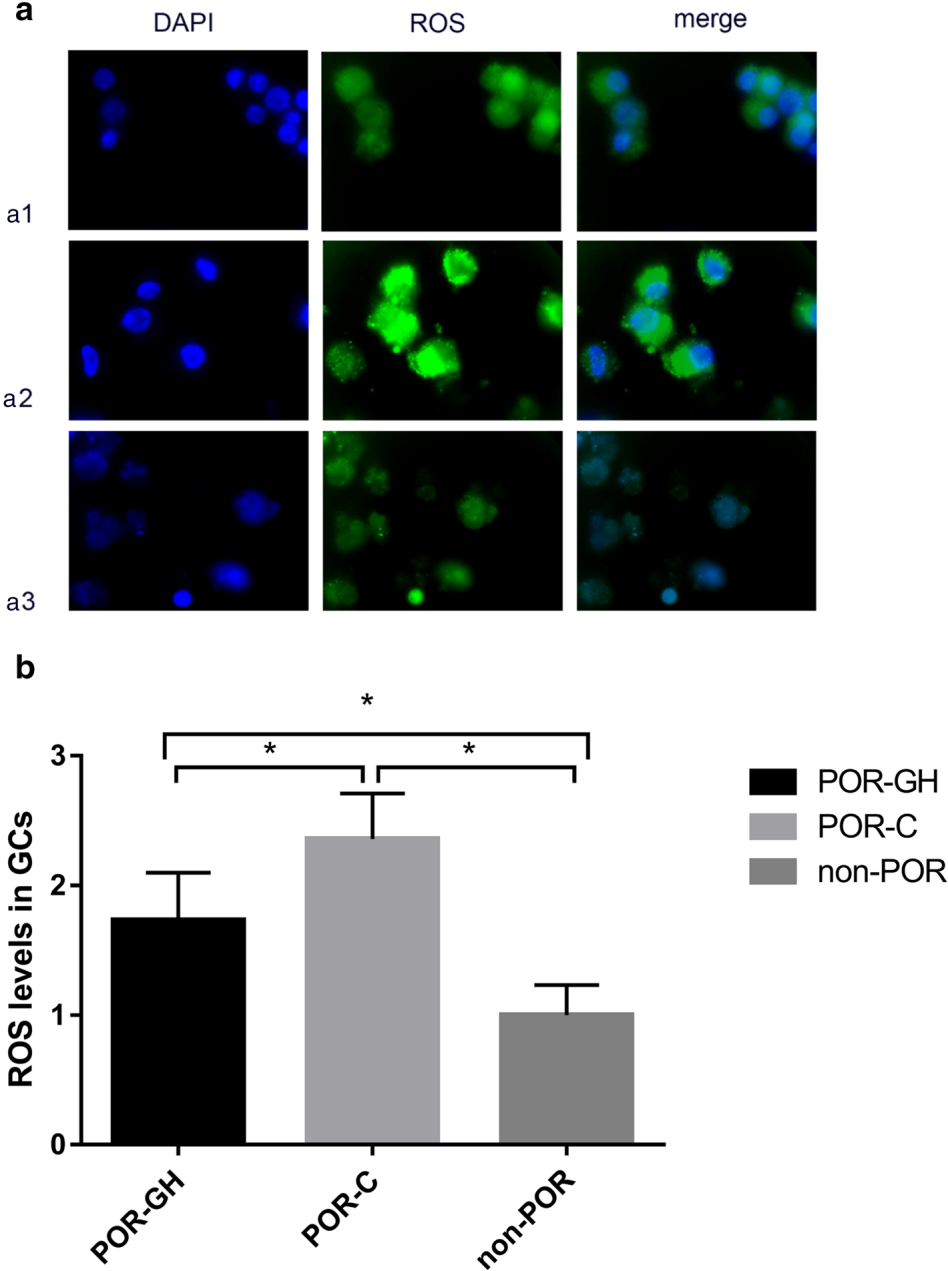
Fluorescence of ROS in GCs of the three groups. **a** The green fluorescence of ROS in GCs was observed under a fluorescence microscope. Cell nuclei were stained with DAPI. a1: POR-GH group, a2: POR-C group, a3: non-POR group. **b** The fluorescence intensity of ROS is expressed as the fold change relative to the control. Measured by spectrophotometry, the fluorescence intensity of ROS (2.36 ± 0.32 vs. 1.00 ± 0.23) was significantly higher in the POR-C group than in the non-POR group (*P* < 0.05). GH significantly lowered ROS intensity in the POR-GH group (1.83 ± 0.38 vs. 2.36 ± 0.32) (*P* < 0.05)

The green fluorescence intensity of ROS in GCs was significantly higher in the POR-GH group and POR-C group than in the non-POR group. Additionally, the fluorescence intensity of ROS (2.36 ± 0.32 vs. 1.00 ± 0.23) was significantly higher in the POR-C group than in the non-POR group (*P* < 0.05). Furthermore, ROS intensity (1.83 ± 0.38 vs. 2.36 ± 0.32) was significantly reduced in the POR-GH group compared with the POR-C group (*P* < 0.05). The fluorescence intensity of ROS is expressed as the fold change relative to the control (Fig. 2).

## Discussion

This study found the existence of an OS state in the FF and GCs of poor ovarian responders undergoing IVF. GH pretreatment lowered the TOS and OSI in FF and intracellular ROS levels but increased the TAC in FF, suggesting that GH may alleviate OS in the FF and GCs of poor ovarian responders. We also found that GH improved the endometrial thickness on trigger day, increased the number of cleaved embryos, and improved embryo quality, the implantation rate and the clinical pregnancy rate. Alleviating OS may be one of the mechanisms by which GH improves oocyte quality and the IVF outcomes of poor ovarian responders.

We found that the levels of MDA, TOS and OSI in FF were higher, but that the levels of SOD and TAC were lower in poor ovarian responders. Numerous studies have demonstrated the OS state in these patients [6, 24, 25]. The TAC represents the ability to eliminate free radicals. Oyawoye et al. reported [26] that the optimum value of the TAC in FF was 0.68 mmol/L for fertilization and subsequent early zygote development, and that the TAC was decreased in women over 37 years old. Similarly, we found that the TAC was 0.71 ± 0.11 mmol Trolox Equiv./L in patients without POR and decreased to 0.42 ± 0.16 mmol Trolox Equiv./L in poor ovarian responders. MDA is one of the end-products of lipid peroxidation. TOS represents antioxidant compounds, and the OSI represents the relative level of OS. Interestingly, we found that GH significantly increased the TAC and decreased the values for TOS and the OSI in FF, with more cleaved embryos and higher-quality embryos. FF plays a critical role in oocyte development; therefore, alleviating OS in FF may improve oocyte quality and IVF outcomes [25, 27]. The above results suggested that GH could improve oocyte quality possibly by suppressing the OS level in FF.

To further assess the OS state in the ovary, we examined the level of ROS in GCs. We found that intracellular ROS levels were significantly higher in poor ovarian responders. GCs are steroidogenic cells surrounding the oocyte. Their metabolic activity is driven by mitochondria and is assumed to provide nutrients and maturation-enabling factors to oocytes [28]. Therefore, GCs play an important role in oocyte development, ovulation, fertilization, and ROS accumulation [29]. Low doses of ROS are important signals for oocyte maturation and ovulation. Under normal physiological conditions, GCs protect oocytes from OS via antioxidant systems, such as SOD and E_2_ [30]. In poor ovarian responders, the antioxidant defence system is suppressed, and more abnormal mitochondrial formation and dysfunction are found in GCs [30]. Furthermore, COS induces ROS production in GCs due to active metabolic and proliferation processes [31]. In general, the above factors trigger GC apoptosis and impair oocyte quality in poor ovarian responders [32]. Here, we found that GH effectively decreased ROS levels in GCs. Our result is consistent with the antioxidant effects of GH in other types of cells, including vascular endothelial cells, myocardial cells and skeletal muscle cells [14–16], suggesting a possible mechanism by which GH could improve oocyte quality.

We found that GH could increase the number of cleaved embryos and embryo quality; however, GH may not affect the number of retrieved oocytes, MII oocytes or fertilized oocytes. GH may improve the quality but not quantity of oocytes by alleviating OS and enhancing mitochondrial function [13]. However, other studies have reported that more overall and fertilized oocytes were achieved by GH treatment, but that no difference was observed in embryo quality [33]. These discrepant results may be due to heterogeneity of the GH protocol, different COS protocols, or inconsistent POR definitions between studies.

In this study, we found that GH improved endometrial thickness in poor ovarian responders. The uterus is a site in which both GH production and GH actions are observed [11]. The glandular cells of the human endometrium and decidual tissue express GHR [34]. A series of studies have confirmed that GH increases endometrial blood circulation, expression of receptivity related genes and cytokines, as result to improve endometrial thickness and receptivity [35, 36]. Endometrial receptivity is an important factor for embryo implantation [36]. Therefore, the effects on endometrial tissue may be another mechanism by which GH affects the IVF outcomes of poor ovarian responders.

In addition to the above benefits, we found that GH could improve the implantation and clinical pregnancy rates of poor ovarian responders. However, another study reported inconsistent results, namely, that GH could not improve the clinical pregnancy rate [37]. We also found that the miscarriage rate was lower in poor ovarian responders pretreated with GH, but the difference was not significant. However, Tesarik J et al. [33] reported that GH significantly decreased the miscarriage rate. These conflicting results can be explained by the discrepancies between the studies.

There were limitations in this study. The sample size was small, and the average age of the patients was 38 years; thus, the results should be analysed and interpreted with caution. Moreover, due to limitations of the study period, we could not evaluate the effect of GH on the live birth rate, which is a more favourable indicator of IVF outcomes. However, whether GH can improve the live birth rate is still controversial [2, 38].

## Conclusion

In conclusion, this study revealed high levels of OS in poor ovarian responders undergoing IVF. GH pretreatment alleviated OS, improved oocyte quality and IVF outcomes in these patients.

## Data Availability

The datasets used and/or analysed during the current study are available from the corresponding author upon reasonable request.
